# Macrophages and hepatocellular carcinoma

**DOI:** 10.1186/s13578-019-0342-7

**Published:** 2019-09-26

**Authors:** Zhiqiang Tian, Xiaojuan Hou, Wenting Liu, Zhipeng Han, Lixin Wei

**Affiliations:** 1 Tumor Immunology and Gene Therapy Center, Eastern Hepatobiliary Surgery Hospital, The Second Military Medical University, 225 Changhai Road, Shanghai, 200438 China; 2 Department of General Surgery, Wuxi People’s Hospital Affiliated Nanjing Medical University, 299 Qingyang Road, Wuxi, 214000 China

**Keywords:** Hepatocellular carcinoma, Macrophage, Tumor microenvironment, Immunotherapy

## Abstract

Hepatocellular carcinoma (HCC) is among the most prevalent and lethal cancers in the human population. HCC is an inflammation-associated cancer caused by different etiological factors. The chronic inflammation leads to continuous cycles of hepatocytes destructive–regenerative process and contributes to HCC initiation and progression. Macrophages play a crucial role in chronic liver inflammation. The tumor microenvironment plays a key role in the progression of HCC. Tumor-associated macrophages are a well-known component of the tumor microenvironment and abundantly infiltrate HCC microenvironment. The roles of macrophages in the development and progression of HCC have been recognized. The deep understanding of macrophages in HCC will be critical for developing effective HCC therapy. Targeting of macrophages might provide novel therapeutic approaches for HCC patients and is an emerging field of interest. This review summarizes the knowledge on the contribution of macrophages in the development and progression of HCC, as well as potential immunotherapy being explored in targeting macrophages.

## Background

Hepatocellular carcinoma (HCC) is one of the prevalent and leading lethal cancers in worldwide [1]. The survival of HCC remains poor despite recent advances in the diagnosis and treatment over the past decades [2]. HCC is an inflammation-associated cancer caused by different etiological factors such as hepatitis virus, non-alcoholic steatohepatitis and alcohol [3, 4]. After chronic liver injury, the damaged liver tissue initiates reparative processes to restore the liver structure and function. The chronic inflammation leads to continuous cycles of hepatocytes destructive–regenerative process and contributes to HCC initiation and progression [5, 6].

The tumor microenvironment, consisting of stromal cells, endothelial cells, immune cells, inflammatory cells, cytokines and extracellular matrix, plays a key role in initiation and progression of HCC [7, 8]. The tumor microenvironment promotes to HCC cells to acquire abnormal phenotypes and recruits immune cells (macrophages, T cells) [9, 10]. Tumor-associated macrophages (TAMs) are a well-known component of the tumor microenvironment, which take part in the cancer progression and metastasis [11]. Macrophages abundantly infiltrate HCC microenvironment and are often associated with poor prognosis of HCC patients. TAMs have important roles in uncontrolled malignant growth by regulating the immune responses to HCC cells and secreting various cytokines. The roles of TAMs in HCC have been recognized, including immunosuppressive function, enhancement of caner invasion and metastasis, angiogenesis, epithelial-mesenchymal transition (EMT) and maintenance of stemness. With this regard, the deep understanding of TAMs in HCC will be critical for developing effective HCC therapy [12]. Targeting of TAMs might provide novel therapeutic approaches for HCC patients and is an emerging field of interest [13].

In this review, we systematically summarize recent findings on the specific characteristic and role of macrophages in HCC progression. Subsequently, we address the potential possibilities of targeting macrophages for HCC immunotherapy.

## Liver macrophages origin and heterogeneity

**Fig. 1:**
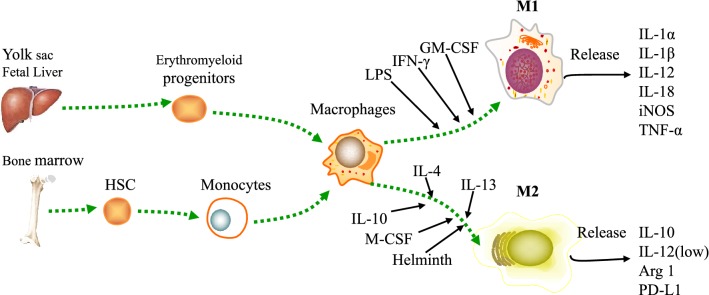
Macrophages origin and heterogeneity. Macrophages are the end cells of the mononuclear lineage. Erythromyeloid progenitors from yolk sac and fetal liver and HSCs from bone marrow develop into the progenitor of macrophages. Macrophages can be induced two distinct polarization phenotypes according to the spectrum of their responses by different microenvironmental stimuli. M1 macrophages exert cytotoxic function by releasing IL-1α, IL-1β, IL-12, IL-18, iNOS, and TNF-α which are induced by LPS, IFN-γ and GM-CSF. M2 macrophages exert anti-inflammatory activities by express low IL-12, high IL-10, arginase 1 and PD-L1 which are induced by IL-4, IL-10, IL-13, M-CSF and helminth. Arg-1, arginine-1; HSCs, hematopoietic stem cells; iNOS, inducible nitric oxide synthase; IFN-γ, interferon-γ; LPS, lipopolysachharide; GM-CSF, granulocyte–macrophage colony-stimulating factor; M-CSF, macrophage colony-stimulating factor; TNF-α, tumor necrosis factor α

Macrophages are the end cells of the mononuclear lineage characterized by phagocytic nature according with mononuclear phagocytic system and arise from myeloid progenitors and circulating monocytes [14]. Several tissue-resident macrophage populations are seeded during waves of embryonic hematopoiesis and self-maintain independently of bone marrow contribution during adulthood [15]. Macrophages are found in all tissues of adult mammals and display incredibly anatomical plastic and functional diversity [16]. Macrophages play a crucial role in the initiation, maintenance, and resolution of inflammation [17]. Macrophages exert phagocytosis, antigen presentation capacity and immune regulation effect by releasing multiple growth factors and cytokines [18] (Fig. 1). Liver macrophages are composed of Kupffer cells and monocytes. Kupffer cells are self-sustaining, non-migratory tissue-resident phagocytes and originate from yolk sac-derived precursors during embryogenesis [19]. Kupffer cells are essential for hepatic and systemic homeostasis, as they are immunogenic in nature and receive signals from the local microenvironment that prompt their functional differentiation [20]. Following their activation by danger signals, Kupffer cells modulate inflammation and recruit immune cells—including large numbers of monocytes—to the liver [21].

However, this definition is inadequate as macrophages have several origins during ontogeny and each of these different lineages persist into adulthood [22]. Other functional classifications of macrophages have included binary classifications that refer to inflammatory states. These include the activated macrophage and alternatively activated macrophage categories, and the derivative M1 and M2 categories for these types of macrophage in the non-pathoen-driven condition [23, 24]. Macrophages can be induced two distinct polarization phenotypes according to the spectrum of their responses by different microenvironmental stimuli, namely, the classically activated M1 and the alternatively activated M2 macrophages [25–27]. The two polarization phenotypes have almost contrarious functions on each other [28].

M1 phenotype is the classically activated macrophage which exerts pro-inflammatory properties, has high antigen presentation and promotes the function of T cells [29]. M1 macrophage exerts cytotoxic function by releasing nitric oxide (NO) and reactive oxygen species (ROS) [30–32]. M1 phenotype is stimulated by microbial products (e.g. lipopolysaccharide) or pro-inflammatory cytokines (e.g. IFN-γ, TNF, or Toll-like receptor ligands). M1 macrophage is characterized by high production of HLA-DR and many pro-inflammatory cytokines like IL-1, IL-6, IL-12, IL-23, TNFα, Type I IFN, CXCL1-3, CXCL-5, and CXCL8-10 [24, 33]. M2 phenotype is the alternatively activated macrophage which exerts anti-inflammatory activities, has immunoregulatory functions and promotes tissue repair [34]. M2 macrophage is induced by Th2 cytokine IL-4, IL-10, IL-13 and glucocorticoids. M2 macrophage is characterized by high production of CCR2, CXCR1, CXCR2, CD163, DC-SIGN, Dectin-1, mannose receptor, scavenger receptor A and B-1 [35]. M2 macrophage expresses high PD-L1 and anti-inflammatory cytokine IL-10, low level of pro-inflammatory cytokine IL-12 [36].

The actual mechanism of macrophage polarization is entirely unclear by now because of the intense crosstalk between macrophage polarization and microenvironment. In recent years, researchers have clarified that macrophage polarization was involved in the progress of cancer. IL-6/STAT3 signaling pathway intermediates the polarization of macrophage during the development of HCC. The inhibition of IL-6/STAT3 pathway turned macrophages into M1-type and enhance the effects of M1 macrophages on HCC cells [37]. There is tentative evidence that macrophage polarization can be switched in response to tumor microenvironment [36]. The deep understanding of the role of macrophages in cancer is of vital importance to development of novel biological target therapies.

## Macrophages as double-edged sword in hepatocarcinogenesis

Chronic liver inflammation which leads to fibrosis and cirrhosis is key in the progression of HCC [38]. Resident hepatic macrophages, termed Kupffer cells, play essential roles in the pathogenesis of chronic liver inflammation. Kupffer cells which predominantly localize within the lumen of the liver sinusoids become activated M1 or M2 macrophages in response to chronic liver injury. Chronic liver inflammation is regulated by a balance of between the two types of Kupffer cells: the anti-inflammatory of M1 macrophages and the pro-inflammatory of M2 macrophages [39]. It seems that M1 macrophages suppresses early HCC tumorigenesis by eliminating the cancer cells as soldiers of adaptive immunity. However, macrophages undergo an M2 to M1 phenotypic shift during the tumor progression of HCC [40, 41]. M2 macrophages promote cancer cells proliferation and invasion by suppressing the adaptive immune system.

Expression of NADPH oxidase 1 by liver macrophages induces the production of inflammatory cytokines and promotes tumor development in mice given injection of diethylnitrosamine (DEN) [42]. CCl4-induced chronic liver injury promotes HCC cells seeding and growth in liver. During the tumors developing, M2 pro-tumor monocyte-derived macrophages infiltrated within the tumors, associated with overexpression MMP-2 and -9 [43]. The proinflammatory myeloid cell surface receptor TREM-1 expressed by Kupffer cells is a pivotal determinant of Kupffer cell, and controls the development and progression of HCC [44]. Hippo signaling is a major oncosuppressive pathway in HCC, and loss it in hepatocytes results in increased macrophage infiltration. Hippo signaling inhibits protumoural microenvironment by suppressing macrophage infiltration through the inhibition of Yap-dependent Mcp1 expression [45]. M2 macrophage activation was associated with chronic hepatitis C infection-induced liver fibrogenesis in a humanized mouse model, and HCV-activated monocytes/macrophages promoted hepatic stellate cell activation [46]. In the liver, steatosis often proceeds cancer formation. Debebe et al. demonstrated that infiltrating macrophages as a key source for steatosis-induced Wnt expression. Wnt/β-catenin is a novel signal produced by infiltrating macrophages induced by steatosis that promotes growth of tumor progenitor cells, underlying the increased risk of liver tumor development in obese individuals [47]. Western diet-induced NASH accelerates HCC progression in a carcinogen initiated model via upregulation of hif-1α mediated IL-10 M2 macrophages polarization [48]. Leukocyte cell-derived chemotaxin-2 (LECT2) is a key player in liver tumorigenesis because LECT2 can controls inflammatory monocytes to constrain the growth and progression of HCC [49]. Increased expression of Six1 in macrophages can stimulate the growth and invasion of HCC by elevating MMP-9 expression [50].

Liver macrophages are also involved in the anti-tumor response of HCC development and progression. NAFLD causes selective intrahepatic CD4^+^ T lymphocyte loss and promotes hepatocarcinogenesis in mouse models and human samples. The interaction of hepatic macrophages with CD4^+^ T lymphocytes prevents hepatocarcinogenesis [51]. The CCL2-CCR2 axis is necessary for clearance of pre-cancerous senescent hepatocytes. Senescent hepatocytes secreted CCL2 in a mouse model of oncogene-induced senescence. CCL2 recruited CCR2^+^ pro-inflammatory monocyte-derived macrophages. Senescence-associated CCL2-CCR2 signaling acts as tumor suppressive in early stages of liver tumorigenesis [52]. The endocannabinoid system exerts key roles in the development of liver fibrosis and fatty liver. But cannabinoid receptor 2 that was predominantly expressed in macrophages seem to have antitumor effects by recruiting CD4^+^ T cells [53].

## Tumor-associated macrophages and hepatocellular carcinoma

### Recruitment of macrophages in HCC tissues

Macrophages were recruited into HCC tissue by up-regulation HMGB1 which was heightened expression by hypoxia via HIF1α. High expression of long non-coding RNA Hox antisense intergenic RNA (HOTAIR) is associated with poor prognosis in HCC. HOTAIR regulates CCL2 expression, which may be involved in the recruitment of macrophages and MDSCs to the tumor microenvironment [54]. Abundant macrophages infiltration is a common feature of malignant tumors and the macrophages around the tumoral region were termed tumor-associated macrophages (TAMs). A growing number of studies showed that TAMs promote tumor cell proliferation, angiogenesis, invasion and metastasis. TAMs represent the predominant type of leukocytes in HCC and play crucial roles during HCC progression. TAMs are located in the stroma of HCC tissue and are polarized toward M2 phenotype [55]. Until now, the contribution of TAMs to the development and progression of HCC has been only partially unraveled. The data of immunogenomic analysis by using The Cancer Genome Atlas (TCGA) show that HCC tissue enrich M2 macrophages and tumor microenvironment in HCC is usually dominated by immune regulatory cells [56, 57].

Increased TAMs are related to poor prognosis in HCC patients after the surgical resection [58]. Yeung et al. showed that M2 macrophages contribute to poor prognosis in HCC and promote tumor growth and invasiveness through CCL22-induced EMT [59]. In many studies, CD68 by immunohistochemical staining is frequently used as an indicator for TAMs. Additionally, CD86 (M1), or CD163 and CD206 (M2) are proposed to distinguish between M1 and M2 macrophages in cancers [60, 61]. Dong et al. showed that low presence of CD86^+^ M1 macrophages and high presence of CD206^+^ M2 macrophages were correlated with aggressive phenotypes of HCC, combined analysis of CD86 and CD206 provided a better indicator for prognosis [62]. HCC occurs more frequently and aggressively in males than in females in the transgenic zebrafish. These tumors of male Zebrafish were more heavily infiltrated with TAMs. This study showed that TAM infiltration was one of the primary factors in the gender disparity of HCC development [63]. ST18 is critical for liver cancer progression and maintenance in a mouse model. TAMs induced epithelial cells expression ST18, ST18 mediated mutual epithelium-macrophage dependency in liver carcinogenesis [64]. In a cohort from Australia, this study showed that soluble CD163 which is a specific macrophage activation marker may predict a rapid HCC progression [65]. The main role of TAMs is to prevent NK cells and other lymphocytes by cytokines of IL-10 and TGF-β [66]. The macrophages from intra-tumoral regions of HCC express CD48 proteins, which induced NK cell dysfunction by blocking CD48 receptor 2B4 on NK cells [67].

Recent investigations in innate immune memory revealed that macrophages could be trained by IGF-1 and IGF-2 with an altered responsiveness [68, 69]. These reprogramming processes of macrophages often occurred during their maturation [70]. Such remodeling of epigenetic landscape could result from a shift in the cellular metabolism of macrophages, since tricarboxylic acid cycle metabolites such as acetyl-CoA, α-ketoglutarate, and succinate are found to play important roles in modulating the enzymes responsible for epigenetic modification [71]. Liver is the major source for IGFs production in vivo. However, the relationship between IGFs-preprogrammed macrophages and HCC remained to be illustrated. Furthermore, the detailed signaling axis connecting metabolic reprogramming, epigenetic modification, and the altered responsiveness still merits further investigation.

### TAMs regulate angiogenesis

The functionally distinct macrophage populations are the characteristics of HCC microenvironment. The CCR2^+^ inflammatory TAM subset accumulates at the highly vascularized HCC and has pro-angiogenic properties or tumor vascularization in fibrotic livers [72]. A nested case–control study based on chronic HBV infection cohort showed that the individuals with HCC outcome had higher serum levels of IL-23. IL-23 which was produced by inflammatory macrophages enhanced macrophage-mediated angiogenesis by upregulation IL-23 receptor expressions on macrophages. IL-23 consequently promoted HCC development after chronic hepatitis B virus infection [73]. The oxidored-nitro domain-containing protein 1 (NOR1) is overexpressed in human HCC tissues associated TAMs promotes M2 alternative polarization. Abnormal expression of NOR1 protein in TAMs contributes to the development of HCC induced by DEN [74]. The chemokine receptor CXCR3 regulates the polarization of TAMs and inhibits cancer growth and angiogenesis of HCC in mice. Macrophages could regulate the expression of CXCR4 via the ERK pathway, which is a novel vascular marker for angiogenesis in HCC tissues. The anti-tumor efficacy of sorafenib combined with zoledronic acid (ZA) was improved by significantly reducing the expression of CXCR4 in vessels [75].

### TAMs promote HCC cells proliferation, invasion and metastasis

IL-6 derived by macrophages can induce EMT of HCC cells, and promote HCC invasion and metastasis [76]. The innate immune response of macrophages to LPS is regulated by miR-101 through targeting dual specificity phosphatase 1 (DUSP1). Macrophage polarization is altered by Sorafenib, and the growth, metastases and EMT driven by TGF-β of HCC in vitro are reduced [77]. miR-28-5p was down-regulated in clinical HCC samples, and its levels were inversely correlated with the number of TAMs and IL-34 expression. IL-34-mediated TAMs infiltration in HCC resulted an miR-28-5p-IL-34-macrophage feedback loop, and the feedback loop modulated HCC metastasis [78]. SPON2 promotes the recruitment of M1 polarization macrophages and inhibits the metastasis of HCC through different integrin-Rho GTPase-Hippo pathways. The study showed that SPON2 is a key factor mediating the immune response against HCC cells growth and migration [79]. Macrophages activating CXCL8 increased the expression of miR-17 cluster in HCC cells, and promoted HCC cells growth and metastasis [80]. Long non-coding RNA cox-2 inhibits immune evasion and metastasis of HCC by inhibiting the polarization of M2 macrophages [81]. Tim-3 expression was increased in TAMs of HCC, and correlated with the poor survival. Tim-3 promotes the development of HCC by enhancing TGF-β-mediated alternative activation of macrophages [82].

miR-98 play a vital role in regulating macrophage polarization by modulating from M2 to M1 in HCC, and suppresses the effects of TAMs on promoting invasion and EMT in hepatocellular carcinoma [83]. The necrotic debris of HCC cells induced potent IL-1β release by TAMs with an M2 phenotype in a hypoxic-inflammatory microenvironment. IL-1β, with its increasing in the local microenvironment, up-regulated the synthesis of HIF-1α in HCC cells by cyclooxygenase-2. And the overexpression of HIF-1α enhanced EMT of hepatoma cells [84]. Yao et al. found that TAMs with an M2 phenotype facilitated the migration and EMT of HCC cells through the TLR4/STAT3 signaling pathway [85]. Aberrant activation of the NTS/IL-8 pathway promoted a pro-oncogenic inflammatory microenvironment and tumor invasion of HCC cells by inducing M2 polarization of TAMs and indirectly promoting EMT [86].

### TAMs affect liver cancer stem cells

Accumulating evidence prove that liver cancer stem cells (LCSCs) account for the substantial heterogeneity and hierarchical organization of liver cancer. LCSCs play a critical role in the recurrence, metastasis, chemotherapy and radiation resistance of HCC. CD44(+) cells isolated from human HCC tissues and cell lines have CSC activities in vitro and in vivo. TAMs produce interleukin 6 and signal via STAT3 to promote expansion of HCC stem cells in human [87]. Fan et al. indicate that the TAMs promote CSC-like properties via TGF-beta1-induced EMT and may contribute to investigate the prognosis of HCC [88]. Li et al. found chronic inflammation-elicited liver progenitor cells (LPCs) can convert to LCSCs, and demonstrated that macrophage-secreted TNF-α triggered chromosomal instability in LPCs through the deregulation of ubiquitin D and checkpoint kinase 2 and enhanced the self-renewal of LPCs through the TNF receptor 1/Src/signal transducer and activator of transcription 3 pathway [89]. Guo et al. demonstrated that tumor-initiating cells (TICs) actively recruit M2 macrophages from as early as the single-cell stage. Activation of the Hippo pathway effector Yes-associated protein (YAP) underlies macrophage recruitment by TICs [90]. TAMs exosomes promote HCC cell proliferation and stem cell properties. Significantly lower levels of miR-125a/b in exosomes and cell lysate isolated from TAMs by using miRNA profiles assay. miR-125a/b inhibits TAMs mediated in CSCs of HCC by targeting CD90 [91]. Chen et al. found that the Wnt/β-catenin pathway was a downstream target of TNF-α and that the Wnt/β-catenin inhibitor ICG-001 partially reversed EMT and attenuated cancer stemness. TNF-α derived from M2 TAMs promotes EMT and cancer stemness through the Wnt/β-catenin pathway in SMMC-7721 cells of HCC [92].

### TAMs modulate therapeutic resistance

Sorafenib, an orally administered multikinase inhibitor, is limited due to individual differences and resistance. A natural CCR2 antagonist from Abies georgei could elevate the number of CD8^+^ T cells in tumors via blocking tumor-associated macrophage-mediated immunosuppression to potentiate the therapeutic effect of sorafenib for liver cancer [93]. Zhou et al. investigated the roles of tumor-associated neutrophils (TANs) in progression of HCC using cell lines and immune cells isolated from patients. The result demonstrated that TANs recruit macrophages and T-regulatory Cells to promote cells growth, progression and resistance to sorafenib of HCC [94]. Oxaliplatin-based chemotherapy is widely used in the treatment of HCC. The density of TAMs in HCC samples was found to associate with the efficacy of transarterial chemoembolization (TACE). TAMs modulate resistance to oxaliplatin by inducing autophagy to avoid apoptosis in HCC [95]. M2 macrophages significantly confer tumor resistance to sorafenib by secreting HGF in a feed-forward manner in HCC [96]. The immunoregulatory mechanism of sorafenib in the treatment of HCC is to induce pyroptosis of macrophages and release NK-cell mediated cytotoxicity [97].

## Macrophages-targeted therapy in hepatocellular carcinoma

**Fig. 2:**
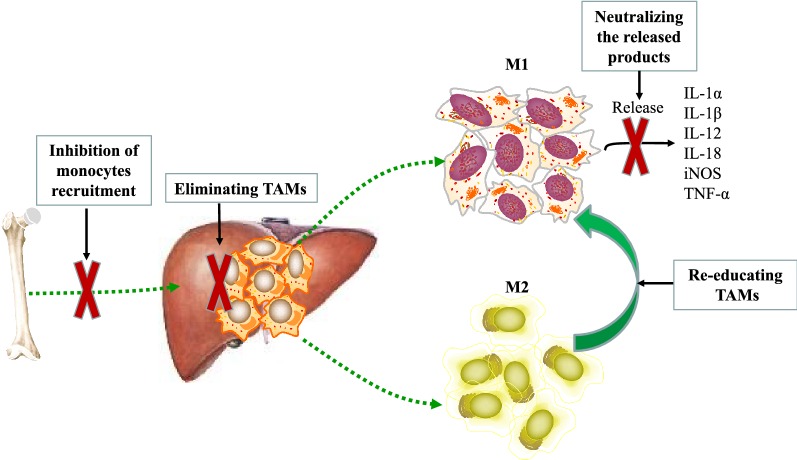
TAMs-targeted strategies in hepatocellular carcinoma. These strategies can be roughly divided into those: (i) inhibition of monocytes recruitment; (ii) eliminating TAMs already present in tumor tissue; (iii) functionally re-educating TAMs polarization; (iv) neutralizing the tumor-promoting products of TAMs

TAMs have a profound influence on the progression of HCC, so there is considerable interest in therapeutic targeting TAMs for HCC immunotherapy. These strategies can be roughly divided into those [98–102]: inhibition of monocytes recruitment, eliminating TAMs already present in tumor tissue, functionally re-educating TAMs polarization, neutralizing the tumor-promoting products of TAMs (Fig. 2). The preclinical of agents targeting TAMs for HCC treatment are listed in Table 1.

**Table 1:** Preclinical of agents targeting TAMs for HCC treatment

Mechanism of action	Compound	Target	Results	References
Inhibition of monocytes recruitment	RDC018 or knockout of CCR2	CCR2 antagonist	Inhibit malignant growth and metastasis, reduces postsurgical recurrence	[103]
	747	CCR2 antagonist	Anticancer properties and potentiated the efficacy of sorafenib in mouse models of HCC	[93]
	CCL2-neutralizing antibody	CCL2	Reduce liver damage, HCC incidence, and tumor burden in mouse models	[104]
	GC33	Glypican-3	Phase I study for advanced HCC	[108, 109]
Eliminating TAMs	Clodrolip or Zoledronic acid		Enhance the inhibitory effect of sorafenib in nude mouse models	[115]
	Zoledronic acid		Enhance the effects of TACE in rat HCC models	[116]
Re-educating TAMs	PLX3397	CSF-1 receptor	Delayed tumor growth and increase in CD8^+^ T cells	[119]
	Baicalin		Inhibition of tumor growth	[117]
	8-Bromo-7-methoxychrysin	CD163	Disrupt the interaction of liver cancer stem-like cells and TAMs	[118]
Neutralizing products of TAMs	Tocilizumab	IL-6 receptor	Inhibit TAM-stimulated activity of human HCC stem cells in vitro and in vivo	[87]

### Inhibition of monocytes recruitment

Inhibition of monocytes recruitment to HCC is an approach to selectively deplete TAMs. CCL2-CCR2 signaling plays a crucial role in TAMs recruitment in most cancer types, and is new target to inhibit the recruitment of monocytes. One of the main sources of CCL2 is Kupffer cells [44]. So, Kupffer cells may participate in the recruitment and education of monocyte-derived macrophages. Li et al. showed that CCL2 is highly expressed and is a prognostic factor in patients with HCC. Blockade of CCL2/CCR2 signaling that inhibits tumor-infiltrating macrophages the switch towards a pro-tumor M2 phenotype, suppresses murine liver tumor growth via activating T cell antitumor immune response [103]. 747, as a natural product from Abies georgei, is an antagonist of CCR2. Yao et al. showed 747 exhibited anticancer properties by elevating tumor-infiltrated CD8^+^ T cells via blocking TAM-mediated immunosuppression and potentiated the efficacy of sorafenib in mouse models of HCC [93]. In addition, Teng et al. showed the tumor-inhibiting effect of a CCL2-neutralizing antibody by reducing the population of inflammatory myeloid cells and inhibiting expression of IL6 and TNFα in a mouse model liver of HCC [104]. MicroRNAs (miRNAs) are another class of small non-coding RNA molecules that can regulate the expression of proteins at the post-transcriptional level. Ectopic expression of miR-26a in a xenograft model of HCC suppressed tumor growth and recruitment of macrophages by down-regulating macrophage colony-stimulating factor (M-CSF) expression through the PI3 K/Akt pathway [105]. Glypican-3 is overexpressed in HCC cells [106] and involve in the recruitment of TAMs in HCC tissues by banding to CCL5 and CCL3 [107]. Antibodies targeting glypican-3 which could inhibit the recruitment of M2-polarized TAMs have shown promise for advanced HCC and have been performed in several Phase I clinical trials [108, 109]. GC33, a humanized antibody against glypican-3, was well tolerated in Japanese patients with advanced HCC [109]. This study showed GPC3 expression in HCC may be associated with the clinical benefit to GC33 [108].

### Eliminating TAMs

As preclinical evidence largely supports the implementation of combinatorial approaches combining targeting TAMs strategies with specific immunotherapy approaches [110]. Sorafenib [111] which is an oral multikinase inhibitor approved for use in HCC inhibited polarized macrophage-induced EMT in human HCC and their migration via the HGF-Met signaling pathway [112]. Zoledronic acid (ZA) can cause a repolarization of the macrophage population by inducing apoptosis specifically in TAMs [113, 114]. Depletion of TAMs by clodrolip or ZA enhanced the inhibitory effect of sorafenib on tumor progression, tumor angiogenesis, and lung metastasis in HCC xenograft nude mouse models [115]. ZA treatment enhanced the effects of TACE through inhibiting TAMs infiltration and tumor angiogenesis in rat HCC models [116].

### Re-educating TAMs

TAM towards M1 phenotype characterizes an immune-competent microenvironment that favors tumor regression. Baicalin, a natural flavonoid present, could block orthotopic growth of implanted HCC in a mouse model. Baicalin initiated TAM reprogramming to M1-like macrophage, and promoted pro-inflammatory cytokines production [117]. 8-Bromo-7-methoxychrysin (BrMC) suppressed the expression of the M2 macrophage marker CD163 and influenced the secretion profile of cytokines of TAMs. BrMC reversed M2 polarization of TAMs induced by liver cancer stem-like cells and may be a potentially novel flavonoid agent to cure HCC [118]. PLX3397, a competitive inhibitor for CSF-1R, could delayed tumor growth murine xenograft models. PLX3397-treated tumors were polarized toward an M1-like phenotype. CSF-1R blockade delayed tumor growth by shifting the polarization rather than the depletion of TAMs [119].

### Neutralizing the tumor-promoting products of TAMs

IL6 which was produce by TAMs during HCC progression promotes expansion of CSCs and tumorigenesis. Tocilizumab is an anti-IL-6 receptor antibody. Tocilizumab was able to inhibit TAM-stimulated activity of human HCC stem cells in vitro and in vivo by blocking IL-6 signaling [87].

## Conclusion

Although macrophages are essential for the normal activity of the immune system, their aberrant regulation are related to HCC. Macrophages abundantly infiltrate HCC microenvironment and have unexpected roles in uncontrolled malignant growth by regulating the immune responses and secreting various cytokines. Recent studies have shown that TAMs play unexpected roles in the development and progression of HCC, including immunosuppressive function, enhancement of caner invasion and metastasis, angiogenesis, inducing EMT and maintenance of stemness. With this regard, the deep understanding of TAMs in HCC will be critical for developing effective HCC therapy. Targeting of TAMs following hepatectomy or liver transplantation might provide novel concepts in adjuvant immunotherapy for HCC patients in the near future. Preliminary data on TAMs-targeted drug interventions suggest that these insights can be successfully translated into new treatment options for HCC patients.

## Data Availability

Not applicable.

## Abbreviations

HCC: hepatocellular carcinoma
TAMs: tumor-associated macrophages
EMT: epithelial-mesenchymal transition
ROS: reactive oxygen species
DEN: diethylnitrosamine
HOTAIR: Hox antisense intergenic RNA
TCGA: The Cancer Genome Atlas
LPCs: liver progenitor cells
LCSCs: liver cancer stem cells
TICs: tumor-initiating cells
TANs: tumor-associated neutrophils
TACE: transarterial chemoembolization
